# Role of maternity waiting homes in the reduction of maternal death and stillbirth in developing countries and its contribution for maternal death reduction in Ethiopia: a systematic review and meta-analysis

**DOI:** 10.1186/s12913-018-3559-y

**Published:** 2018-10-01

**Authors:** Tegene Legese Dadi, Bayu Begashaw Bekele, Habtamu Kebebe Kasaye, Tadesse Nigussie

**Affiliations:** 1 Department of public health, Collage of Health Science, Mizan-Tepi University, Tepi, Ethiopia; 2 Department of midwifery, Collage of Health Science, Wollega University, Nekemte, Ethiopia; 3 Mizan Teferi, Ethiopia

**Keywords:** Maternity waiting homes, MWHs, Maternal mortality, Stillbirth, Developing countries, Ethiopia, Maternal death

## Abstract

**Background:**

Every family expect to have a healthy mother and new born baby after pregnancy. Especially for parents, pregnancy is a time of great anticipation. Access to maternal and child health care insures safer pregnancy and its outcome. MWHs is one the strategy. The objective was to synthesize the best available evidence on effectiveness of maternity waiting homes on the reduction of maternal mortality and stillbirth in developing countries.

**Methods:**

Before conducting this review non-occurrences of the same review is verified. To avoid introduction of bias because of errors, two independent reviewers appraised each article. Maternal death and stillbirth were the primary outcomes. Review Manager 5 were used to produce a random-effect meta-analysis. Grade Pro software were used to produce risk of bias summary and summary of findings.

**Result:**

In developing countries, maternity waiting homes users were 80% less likely to die than non-users (OR = 0. 20, 95% CI [0.08, 0.49]) and there was 73% less occurrence of stillbirth among users (OR = 0.27, 95% CI [0.09, 0.82]). In Ethiopia, there was a 91% reduction of maternal death among maternity waiting homes users unlike non-users (OR = 0.09, 95% CI [0.04, 0.19]) and it contributes to the reduction of 83% stillbirth unlike non-users (OR = 0.17, 95% CI [0.05, 0.58]).

**Conclusion:**

Maternity waiting home contributes more than 80% to the reduction of maternal death among users in developing countries and Ethiopia. Its contribution for reduction of stillbirth is good. More than 70% of stillbirth is reduced among the users of maternity waiting homes. In Ethiopia maternity waiting homes contributes to the reduction of more than two third of stillbirths.

**Electronic supplementary material:**

The online version of this article (10.1186/s12913-018-3559-y) contains supplementary material, which is available to authorized users.

## Background

Pregnancy is a time of great anticipation to have healthy baby and mother after pregnancy. Experiencing maternal death and stillbirth in the final stages of pregnancy is unfortunate for families [1]. To make pregnancy and its outcome safer, every woman should have access to maternal and child health care services during pregnancy, delivery and a period after delivery [2]. As the recommendation of WHO, improvement of obstetric care services can be accomplished by the following three methods: “1. Providing health care services for mothers in need - “flying squads””, “2. Making favourable condition for mothers who need medical services - emergency transport”, and “3. Making health care services more accessible to women [2]. The third solution, needs extensive expenditure on health service, including expanded skilled human resources, which is difficult for developing countries [2, 3].

Many women in low and middle income countries face the challenges of inaccessibility of obstetric care in rural and urban areas. Even where services are available the facilities are unequipped [2]. Rural women are 3.572 times more likely to die because of pregnancy or delivery than women who came from urban areas with 95% CI (1.001, 6.726). This might be rural females do not utilize maternal health services due to different reasons. As a result they may face high obstetric complications [4].

To minimize these problems, developing countries used MWH (maternity waiting homes) as an alternative to increase accessibility of obstetric care services. MWH are homes built in the compound or near to health facilities that provides standard medical and emergency obstetric care services. This is the easiest way to decrease the complication related to child birth through avoiding the second delay. It decreases barriers, which includes: distance, geography, transport, cost of transport and communication between referral points, that inhibit access to service, [2, 3, 5].

Globally low and middle income countries contribute about 99% of maternal death in 2015, from this sub-Saharan Africa accounts for 66% of maternal death which is discriminately high [6]. In 2015 the estimated global stillbirth was 18·4 per 1000 births, it was decreased by 25% from that of 2000. In the same year, in sub-Saharan Africa it was decreased by 19%, which was low progress [6]. Low and middle income countries contributes 98% of stillbirths; sub-Saharan Africa and South Asia share about 77% [7]. Majority of losses related to pregnancy and child birth can be prevented through providing high quality and evidence based services [1].

According to EWEC technical workstream working group, by 2030 from 2010, all countries have to decrease MMR (maternal mortality rate) by a minimum of two thirds. In 2030 the target for the globe is < 70 per 100,000 live births, but no country have to have a MMR of more than 140 per 100,000 live births. By 2030, the maximum stillbirth’s rate is <= 12 per 1000 live births for every country [8, 9]. To achieve these targets each country has to work towards minimizing barriers of accessing quality maternal and child health care services. Therefore, planners should analyse their contextualised problems, researching available services, and implementation of rational framework for prioritizing and scaling up of essential services [8, 9].

### Research gaps identified

There is one scoping review by Julie M. Buser et al. which shows new born outcomes of maternity waiting homes. This review doesn’t appraise the quality of evidence and doesn’t provide evidences of effectiveness of MWHs, it only narrate the available researches [10]. The systematic review on the Cochrane Review published in 2012 didn’t include any randomized controlled studies and did not perform meta-analyses. It provides limited information on the potential benefit of MWH [11]. Thus, it is essential to prove efficacy of MWH through systematic reviews and meta-analysis.

In addition to the above reasons, the WHO 2015 endorsements on “health promotion interventions for maternal and newborn health states that there is a research gap on identifying efficacy of MWH.” So there is a need for a study whether the MWH effectively improves birth outcomes or not [3].

### Objective of this review

The objective of this review was to systematically identify, appraise and synthesize the best available evidence on effectiveness of MWHs on the reduction of maternal mortality and stillbirth in developing countries.

### Research questions

Is constructing MWHs in developing countries is effective in decreasing maternal death and stillbirth?

How much MWHs kick in to the reduction of maternal death and stillbirth in Ethiopia?

## Methods

### Search strategy

Prior to conducting this review different databases, which publish reviews, was searched in 2017 G.C to verify the absence of antecedent reviews or protocols. The searched databases were: the Joanna Briggs Institute Database of Systematic Reviews and Implementation Reports (JBI- DSRIR), the Cochrane Database of Systematic Reviews, the Campbell Collaboration library, the National Health Centre Reviews and Dissemination databases, Evidence for Policy and Practice Information (EPPI-Centre). The search was using keyword and index search terms: Maternity waiting homes and maternity waiting areas with maternal death and stillbirth.

Except one review by Van Lonkhuijzen L. et al. from Cochrane Database of Systematic Reviews, no review is conducted as well as no protocol is developed. The objective of the study by Van Lonkhuijzen L. et al. was to see the effect of MWHs on maternal outcome using only clinical trials study. This review did not get any clinical trials and no result is provided by the review [11]. A scientific literature search from AJOL, PubMed, Google scholar, EMBASE, Ovid and Scopus databases was conducted from March – June 2017 GC using different search terms, which is listed in Additional file 1. Gray literatures were searched from Google and Google scholar, the largest store of gray literatures.

In addition, literatures were searched from research gate. For additional studies that may have been missed in the electronic search, cross reference was undertaken using the reference lists of all identified articles. Articles identified from variety sources were assessed for importance as per the objective of the study. All authors participated in searching each database. An abstract and full report was captured that meet the inclusion criteria.

### Inclusion and exclusion criteria

The criteria for inclusion in the review includes quantitative research reports on effectiveness of MWHs on the decreasement of maternal death and stillbirth. In addition to the above mentioned, articles performed in developing countries and published in the English language are the criteria. We don’t have a limitation on starting time of paper publication. Articles were excluded, if they are pure qualitative research, if data not presented for a comparator, if no data presented for the desired outcome, editorials and short commentaries.

### Selection of articles

Study selection was conducted by all authors independently. The selection process was first started by avoiding duplicates using Mendeley Desktop reference manager. Next reviewing the titles and abstracts of all collected literatures were performed. Literatures with unrelated title and abstract were excluded. All relevant articles were considered for full review. When there were disagreements in the grouping of articles, decision was made by discussion and by reviewing the articles together.

Besides above mentioned inclusion criteria’s, each paper that meet the inclusion criteria are critically reviewed by two independent reviewers for a single stud for methodological validity. It is appraised by appraisal instruments from the Joanna Briggs institute meta-analysis of statistical assessment and review instrument (JBI-MAStARI) (Additional file 1).

### Study outcomes

#### Primary outcomes

Maternal death

Stillbirth

#### Secondary outcomes

Neonatal mortality

Parity

### Assessment of quality of evidence across studies

Grade Pro software (Grade Pro 2016) is used to measure the quality across studies and to summarize findings. It has four levels: high, moderate, low or very low. Observational studies were categorized as low quality. But can be upgraded to moderate quality depending on the types of studies and factors that can increase the quality level. The factors that increase the levels are: large effect, if all plausible confounding would lead to an underestimation of the effect and if there is a dose-response gradient.

### Data extraction

We extracted results of above-mentioned outcomes using data extraction tool from JBI-MAStARI (Additional file 1). All results were taken out by two independent reviewers to avoid extraction error.

### Data analysis

Review Manager 5 is used for statistical analysis and Grade Pro software (Grade Pro 2016) is used to produce a summarized findings. Random-effect meta-analysis was performed to pool the odds ratio (OR) of the outcomes of maternal death and stillbirth. The assumption for random effect meta-analysis is from a range of populations in which the effect size varies and our goal is to summarize this range of effects. The conditions of the fixed effect model are not met, since the true effect size for all studies is identical, and the only reason the effect size varies between studies is random error. Each study is estimating an effect size for its unique population, and so each must be given appropriate weight in the analysis. We summarized the effect in terms of OR with their 95% CI, risk difference (RD) and anticipated absolute effects. Forest plot containing OR, 95% confidence intervals (CI), *P* value, effect size, and, heterogeneity (I^2^) were constructed. *P* value <= 0·05 was considered statistically significant. Sensitivity analysis or subgroup analysis is performed for meta-analysis incorporating more than two studies when the heterogeneity (I^2^) is above 60% by removing outlier study. The forest plot is presented for each subgroup analysis.

## Result

**Fig. 1:**
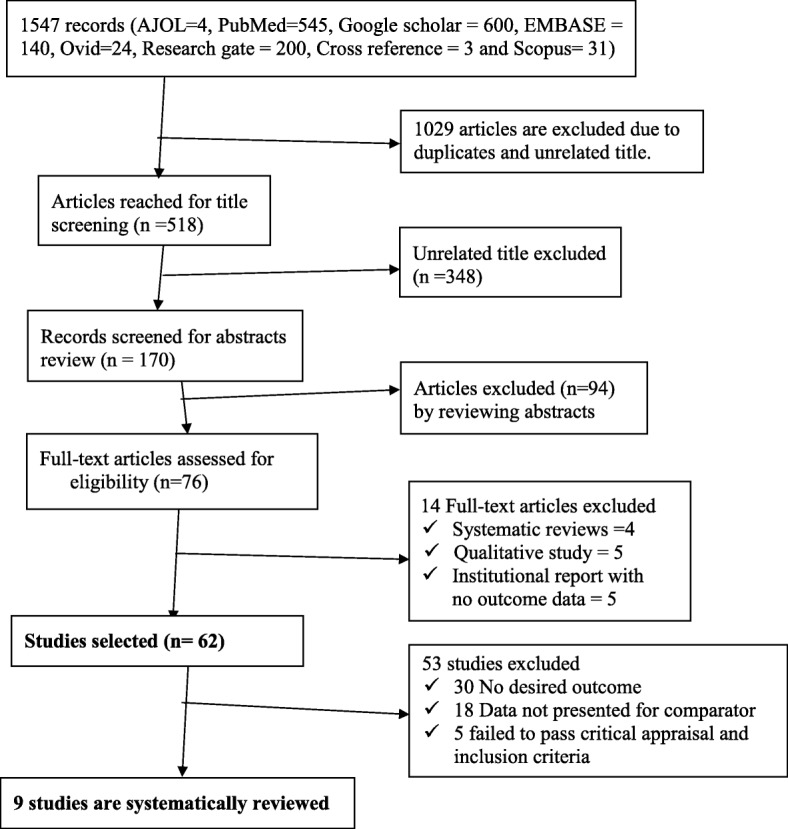
flow chart of study selected

A total of 1547 articles were identified through databases searching. Of these, 1029 articles were excluded as duplicates and by observation of titles. Seventy six articles were identified for full text review, 67 of them are excluded due to not meeting the inclusion criteria. Nine studies were included in the review (Fig. 1).

### Summary of findings and description of included studies

Nine studies are included to assess the primary and secondary outcomes. Parity is reported by 6 studies, but they reported in a different classification. Early neonatal mortality is reported by two studies. Age of mother is reported by only Singh Kavita et al. (Table 1).

**Table 1:** summary of included studies

Author	Set up	Study design	Outcome measure	Proportion for MWHs users	Proportion for non MWHs users
J.M. Tumwine et al. 1996 [17]	Zimbabwe	Retrospective cohort	Stillbirth	3/280	16/773
			Early neonatal mortality	4/280	7/773
			Maternal death	1/277	3/757
			Parity = 0	89/280	215/773
			1–4	121/280	367 /773
			> = 5	70/280	191 /773
P.Millard et al. 1991 [18]	Zimbabwe	Comparative study	Stillbirth	9 /486	14 /336
			Early Neonatal mortality	8 /486	14 /336
			Parity = 0	123 /486	80 /336
			1–3	204 /486	137 /336
			4–6	121 /486	86 /336
			> = 7	36 /486	30 /336
Lonkhuijzen Luc van et al. 2003 [19]	Zambia	Retrospective cohort	Maternal death	0 /218	1 /292
			Parity =0	54 /218	31 /292
			> 6	12 /218	8 /292
Andemichael Ghirmay et al. 2009 [20]	Eritrea	Before and after study	Maternal death	0 /866	5 /266
Jody R Lori et al. 2013 [21]	Liberia	Prospective cohort	Maternal death	3 /248	12 /255
Singh Kavita et al. 2017 [22]	Malawi	Cross sectional	Stillbirth	3 /255	1 /287
			Parity =1	115 /249	104 /288
			2–3	73 /249	112 /288
			> = 4	61 /249	68 /288
			Age = 15–19	62 /259	45 /288
			20–34	164 /259	216 /288
			> = 35	33 /259	26 /288
Poovan Pia et al. 1990 [23]	Ethiopia	Retrospective cohort	Maternal death	0 /142	13 /635
			Stillbirth	4 /142	161 /635
J Kelly et al. 2010 [24]	Ethiopia	Retrospective cohort	Maternal death	6 /6805	187 /17343
			Stillbirth	120 /6805	3316 /17343
			Parity =0	193 /615	525 /1099
			1–3	294 /615	366 /1099
			> = 4	118 /615	177 /1099
D.Chandramohan et al. 1994 [25]	Zimbabwe	Retrospective cohort	Maternal death	1 /1573	2 /2915
			Primiparas	661 /1573	1108 /2915
			Parity > 6	110 /1573	146 /2915

The quality of our evidence across studies is moderate. All of studies are consistent that uses of MWHs have a better outcome in the prevention of maternal death and stillbirth. The result of anticipated absolute effect shows, the risk of maternal death with MWHs is 1 per 1000 live births. However the risk for pregnant females who don’t use MWHs is 10 per 1000 live births. There is a great disparity in the occurrences of stillbirth among MWHs users and non-users. Among the users’ risk of stillbirth is 17 per 1000 live births, but for non-users risk raises by more than ten times (181 per 1000 live births) (Table 2).

**Table 2:** Quality assessment and summary the findings for the primary outcomes in developing countries

Outcomes	№ of participants (studies)	Quality of the evidence (GRADE)	Anticipated absolute effects		
			Risk with MWH	Risk with Non MWH	Risk difference with MWH
Maternal death	32,592 (7 observational studies)	⨁⨁⨁◯ MODERATE	1 per 1000 (11/10,129)	10 per 1000 (223/22,463)	9 fewer per 1000 (10 fewer to 8 fewer)
Stillbirth	27,342 (5 observational studies)	⨁⨁⨁◯ MODERATE	17 per 1000 (139/7968)	181 per 1000 (3508/19374)	164 fewer per 1000 (166 fewer to 159 fewer)

GRADE Working Group grades of evidence

High quality: We are very confident that the true effect lies close to that of the estimate of the effect

Moderate quality: We are moderately confident in the effect estimate: The true effect is likely to be close to the estimate of the effect, but there is a possibility that it is substantially different

Low quality: Our confidence in the effect estimate is limited: The true effect may be substantially different from the estimate of the effect

Very low quality: We have very little confidence in the effect estimate: The true effect is likely to be substantially different from the estimate of effect

Anticipated absolute effects result for studies retrieved from Ethiopia shows maternal death among non MWHs users (11 per 1000 live births) is more than ten times higher than MWHs users (0.86 per 1000 live births). The risk of stillbirth is 3.6 times higher among MWHs non users than users (Table 3).

**Table 3:** Quality assessment and summary the findings for the primary outcomes in Ethiopia

Outcomes among MWH and Non MWH users	№ of participants (studies)	Anticipated absolute effects^*^ (95% CI)			Quality of the evidence (GRADE)
		Risk with Non MWH	Risk with MWH	Difference	
Maternal death in Ethiopia	24,925 (2 observational studies)	11 per 1000	0.86 per 1000 (0 to 2)	10.14 fewer (9 fewer to 8 fewer)	⨁⨁⨁◯ MODERATE
Stillbirth in Ethiopia	24,925 (2 observational studies)	65 per 1000	18 per 1000 (16 to 22)	47 fewer (49 fewer to 43 fewer)	⨁⨁⨁◯ MODERATE

### Maternal death

#### Effect of MWHs on maternal death in developing countries

**Fig. 2:**
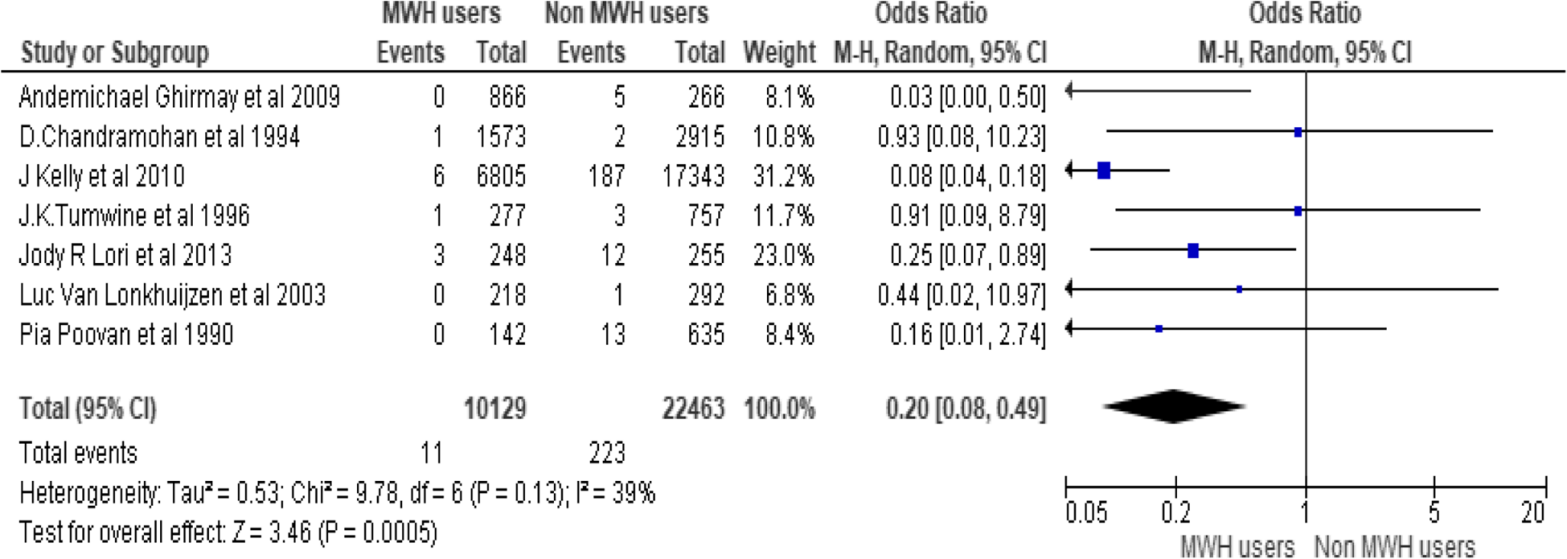
Effect of MWHs on maternal death in developing countries

Seven studies including a total of 32,592 participants reported the occurrence of maternal death among MWH users and non-users. There are 11 maternal deaths out of 10,129 MWHs users and 223 maternal deaths out of 22,463 MWHs non users. MWH user mothers are 80% less likely to die than non-users (OR = 0. 20, 95% CI [0.08, 0.49]), I^2^ = 39%, (*P* < 0.00001) (Fig. 2).

#### Effect of MWHs on maternal death in Ethiopia

**Fig. 3:**
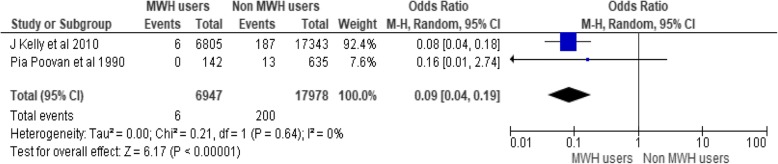
Effect of MWHs on maternal death in Ethiopia

There are three studies conducted in Ethiopia incorporating a total of 24,925 participants (6947 MWH users and 17,978 non users). Only 6 maternal death occurred among users of MWH but, 200 deaths occurred among non-users. There is a 92% reduction of maternal death among MWHs users as compared to non-users (OR = 0.09, 95% CI [0.04, 0.19]), I^2^ = 0%, (*P* < 0.00001) (Fig. 3).

### Stillbirth

#### Effect of MWHs on stillbirth in developing countries

**Fig. 4:**
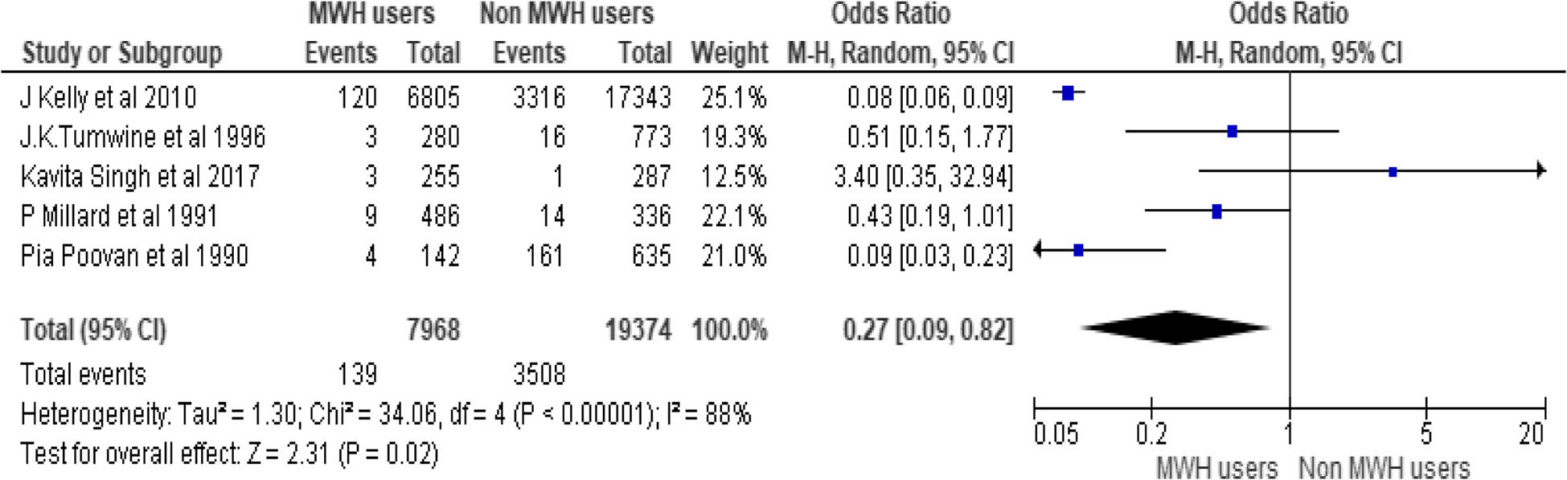
Effect of MWHs on stillbirth in developing countries

To assess effects of MWHs on stillbirth five studies are pooled, including 27,342 participants (7968 MWHs users and 19,374 non users). Occurrences of stillbirth among non-users are more than ten times as compared to non-users. There is 73% less occurrence of stillbirth among users (OR = 0.27, 95% CI [0.09, 0.82]), Chi^2^ = 34.06, df = 4(P < 0.00001); I^2^ = 88% (Fig. 4).

**Fig. 5:**
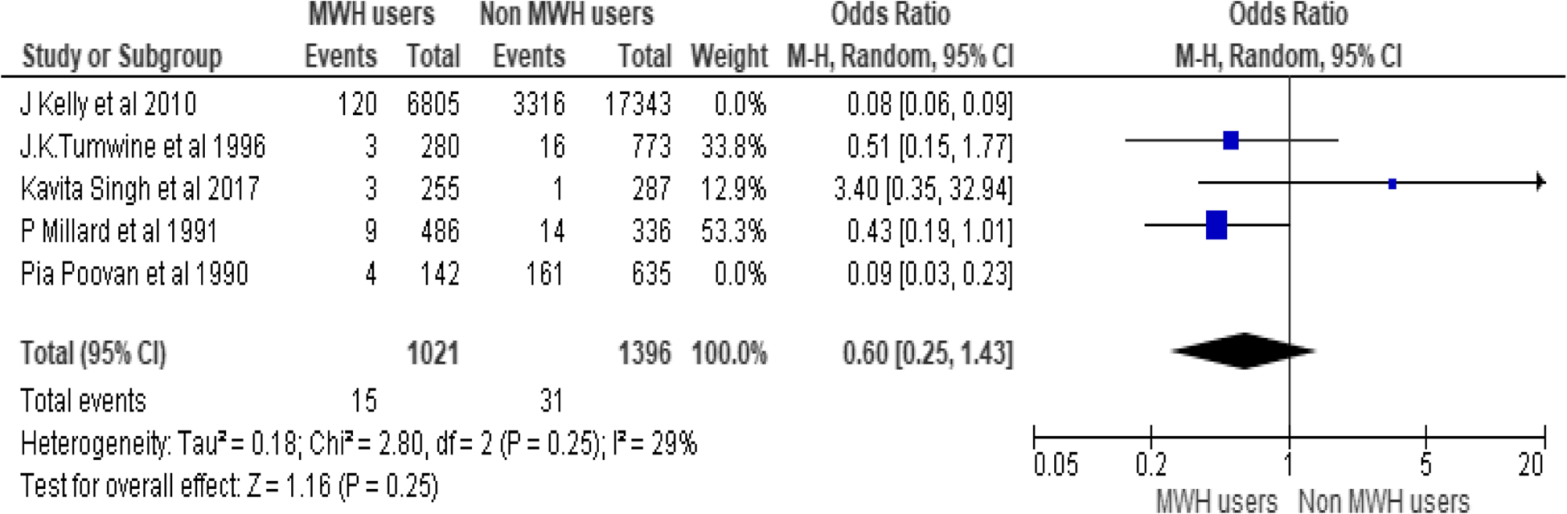
Effect of MWHs on stillbirth in developing countries removing the effect of JKelly et al. 2010 and Pia Poovan et al. 1990

The heterogeneity is large (I^2^ = 88%). In order to treat this heterogeneity, Subgroup analysis is conducted by removing outlier studies. The subgroup analysis is performed by removing the effect of J Kelly et al. 2010 and Pia Poovan et al. 1990 the heterogeneity becomes 29%. However the odds ratio is not significant (Fig. 5).

#### Effect of MWH on stillbirth in Ethiopia

**Fig. 6:**
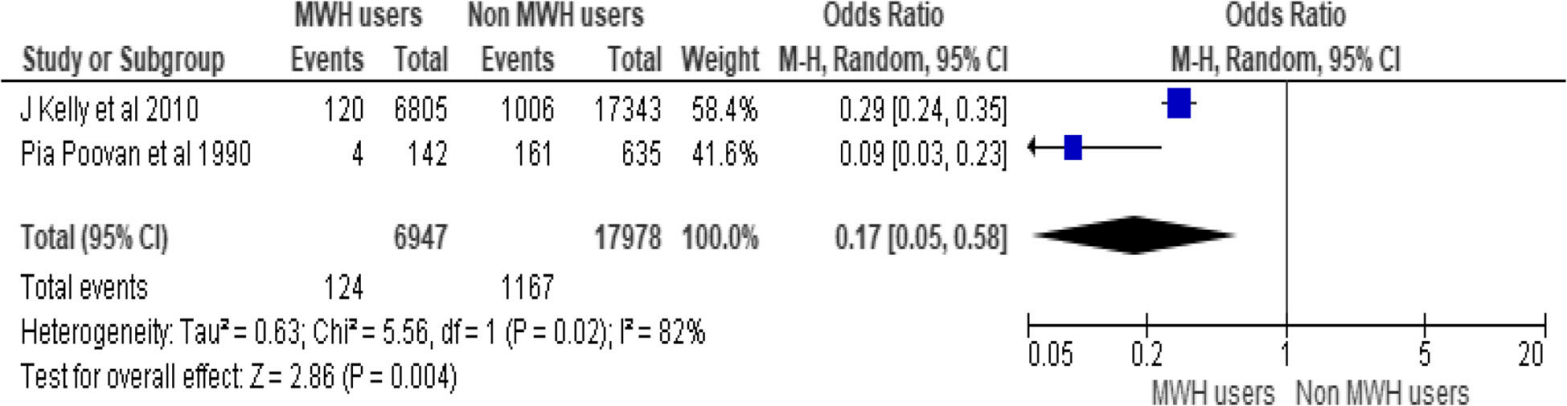
Effect of MWHs on stillbirth in Ethiopia

To observe the contribution of MWHs on reduction of stillbirth only two studies are included in the analysis of fixed effect meta-analysis. These studies incorporate a total of 24,925 participants. MWHs utilization contributes to the reduction of 83% stillbirth as compared to non-users (OR = 0.17, 95% CI [0.05, 0.58]), Chi^2^ = 5.56, df = 1(*P* < 0.02); I^2^ = 82% (Fig. 6).

Even though the difference is significant the heterogeneity among the study is 82% (I^2^ = 82%). The reason for this heterogeneity is the number of participants included. JKelly et al. 2010 includes more than 20 times of participants than the other study. This makes JKelly et al. 2010 as outlier or vice versa.

### Secondary outcomes

#### Early neonatal mortality

**Fig. 7:**
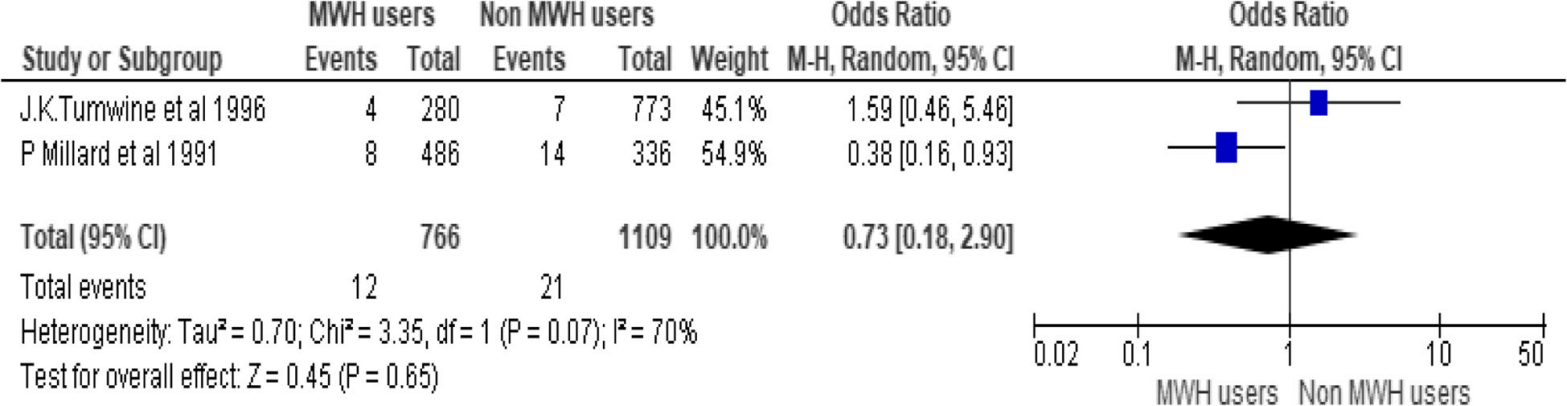
Effect of MWHs on early neonatal mortality in developing countries

There are only two studies that include early neonatal mortality as an outcome in addition to the primary outcomes. They include a total of 1875 participants. Both of the studies are not in line whether MWH utilization reduces early neonatal mortality or not. Their aggregate effect is also not significant. But a higher proportion of early neonatal death occurred among non-users of MWHs than users (Fig. 7).

### Age of mother

Age of mother is reported only by Singh Kavita et al. 2017. Most of users of MWHs (63.4%) are mothers in the age group of 20–34. From females who are not users of MWHs 75% of them are in between 20 and 34 (Table 4).

**Table 4:** maternal age and parity distribution among MWHs users and non-users

Author	Set up	Study design	Outcome measure	Proportion for MWHs (%)	Proportion for non MWHs (%)
J.M. Tumwine et al. 1996 [17]	Rural Zimbabwe	Retrospective cohort	Parity = 0	89/280 (31.8)	215/773 (27.8)
			1–4	121/280 (43.2)	367/773 (47.5)
			> = 5	70/280 (25)	191/773 (24.7)
P.Millard et al. 1991 [18]	Rural Zimbabwe	Comparative study	Parity = 0	123/486 (25.4)	80/336 (23.8)
			1–3	204/486 (42)	137/336 (40.8)
			4–6	121/486 (25)	86/336(25.6)
			> = 7	36/486 (7.6)	30/336 (8.8)
Lonkhuijzen Luc van et al. 2003 [19]	Rural Zambia	Retrospective cohort	Parity =0	54/218 (24.8)	31/292 (10.6)
			> 6	12/218 (5.5)	8/292 (2.7)
Singh Kavita et al. 2017 [22]	Malawi	Cross sectional	Parity =1	115/249 (46.2)	104 /288 (36.1)
			2–3	73/249 (29.3)	112/288 (38.9)
			> = 4	61/249 (24.5)	68 /288 (23)
			Age = 15–19	62/259 (23.9)	45/288 (15.6)
			20–34	164/259 (63.4)	216/288 (75)
			> = 35	33/259 (12.7)	26/288 (9.4)
J Kelly et al. 2010 [24]	Ethiopia	Retrospective cohort	Parity =0	193/615 (31.4)	525 /1099 (47.8)
			1–3	294/615 (47.8)	366/1099 (33.3)
			> = 4	118/615 (19.2)	177 /1099 (16.1)
D.Chandramohan et al. 1994 [25]	Ethiopia	Retrospective cohort	Primiparas	661/1573 (42)	1108/2915 (38)
			Parity > 6	110/1573 [7]	146/2915 [5]

### Parity

Six studies reported parity distribution of study participants. Each of them reported in different ways so unable to pool the results together. The two studies from Zimbabwe (by J.M.Tumwine et al. and P.Millard et al) shows when females’ parity is > = 1 they less likely utilize MWHs. But they most likely use when they don’t have children (parity = 0). In addition, study from Zambia (by Lonkhuijzen Luc van et al) shows females more likely use MWHs when they don’t have children. In contrast to the above result from Zimbabwe, study from Ethiopia (by J.Kelly et al) shows females most likely use when they have children (parity > = 1) (Table 4).

## Discussion

MWH is one part of the strategy to boost uptake of maternal health services in developing countries by overcoming demand-side barriers. It avoids problems created due to long distance from health institutions. Since they can stay and await labour for high-risk pregnant women [3, 12]. This meta-analysis depicts MWHs has a great contribution in decreasing maternal death and stillbirth rate in developing countries. The fixed effect meta-analysis shows MWHs have 80% contribution for reduction of maternal mortality among users (OR = 0. 20, 95% CI [0.08, 0.49]). This result might be underestimated since most of the time high-risk pregnant women are admitted in MWHs. The implication, for developing countries is that further expansion of MWHs an alternative best solution for rural areas.

Ethiopia reduced maternal mortality by 71.8% from 1250 in 1990 to 353 per 100,000 live births in 2015. Studies on health care institutions also show there is decreasing trend of maternal mortality [6, 13, 14]. Increasing accessibility of health services has its own contribution to the reduction. The meta-analysis depicts 91% reduction of maternal death among MWHs users unlike non-users (OR = 0.09, 95% CI [0.04, 0.19). MWHs have their own contribution by decreasing second delay or by bridging high-risk pregnant women living far away from health institutions [6, 12].

The highest burden of stillbirth is found in low and middle- income countries (98%). Sub-Saharan Africa and southern Asia regions accounts for 77% of stillbirth. Its reduction is lowest in sub-Saharan Africa (1·4%) [7]. The causes for the high rates of stillbirth is due to poor maternal health care services [15]. There is a great disparity on the occurrences of stillbirth among MWHs users and non-users. This meta-analysis tells us that MWHs have an amicable role to reduce stillbirth. There is 73% less occurrence of stillbirth among MWHs users (OR = 0.27, 95% CI [0.09, 0.82]) as compared to non-users. This finding implies that, constructing MWHs or strengthening its construction as a strategy to reduce stillbirth is effective mode of interventions.

Ethiopia is the fifth country in the world by having 97, 000 stillbirths [7]. MWHs utilization contributes to the reduction of 83% of stillbirth unlike non-users (OR = 0.17, 95% CI [0.05, 0.58]). As above mentioned, MWHs are crucial as a prevention modalities.

Low and middle income countries contribute 99% of neonatal deaths; about half of them occur at home. The first week of life covers three fourth of death. The highest risk of death is on the first day of life [16]. There are only two studies pooled together. Study by P.Millard et al. shows MWHs use reduce early neonatal mortality. The other study is not significant. Their aggregate effect is not significant. But a higher proportion of early neonatal death occurred among non-users of MWHs than others.

These findings suggest that construction of maternity waiting home near health facility is one of the effective strategies to break phase II delays or transportation delays in developing countries.

### Limitation of study

This systematic review and meta-analysis is conducted incorporating observational studies only. Literatures written by other than English is not included. Literatures included from Ethiopia in this review is conducted in only one place at different times. It does not represent the whole country. There might a possibility of publication bias since published literatures written in English is included.

## Conclusion

Having the abovementioned limitation of the study: Maternity waiting home contributes almost 80% for the reduction of maternal death among users in developing countries and Ethiopia as per this review. Its contribution for reduction of stillbirth is good. More than 70% of stillbirth is reduced among the users. In Ethiopia MWHs contributes to a reduction of more than two third stillbirths among users. The effect of MWHs on early neonatal mortality among MWHs users and non-users is not significant. But, a higher proportion of deaths occur among non-users. The major implication of this review is MWHs are effective for the accomplishment of sustainable development goals related to maternal and child health. The authors recommend further review as a primary outcome to confirm whether MWHs is effective in reducing early neonatal death or not. In addition randomized controlled trial study is the gold standard method to prove the effectiveness of maternity waiting home.

## Additional file

Additional file 1: This file contain Index and mesh terms, the search strategy used in different databases, critical appraisal formats, and data extraction format (DOCX 645 kb)

## Abbreviations

AOR: Adjusted odds ratio
CI: Confidence interval
DHS: Demographic and health survey
EDHS: Ethiopian demographic and health survey
EPPI-Centre: Evidence for Policy and Practice Information
HTA: Health Technology Assessment
JBI- DSRIR: Joanna Briggs Institute Database of Systematic Reviews and Implementation Reports
JBI-MAStARI: Joanna Briggs institute meta-analysis of statistical assessment and review instrument
MWHs: Maternity waiting homes
OR: Odds ratio
SSA: Sub – Saharan Africa
WHO: World health organization
