# Supplementary material for: Role of maternity waiting homes in the reduction of maternal death and stillbirth in developing countries and its contribution for maternal death reduction in Ethiopia: a systematic review and meta-analysis
Source: BMC Health Serv Res. 2018 Oct 1;18:748. doi: 10.1186/s12913-018-3559-y (PMC6167854; doi:10.1186/s12913-018-3559-y)

**Additional file: search strategy**

**Phrases used to search articles from google scholar, AJOL, research gate, Scopus and EMBASE**

Maternity waiting home

Maternity waiting areas

Maternity waiting villages

Maternity waiting shelters

Maternity waiting dormitories

Maternity waiting home AND maternal death OR stillbirth

Maternity waiting home AND maternal mortality OR stillbirth

Maternity waiting areas AND maternal death OR stillbirth

Maternity waiting areas AND maternal mortality OR stillbirth

**Search terms for PubMed**

lang: English

MeSH Terms: Infant, Newborn; female; humans

"Infant, Newborn"[Mesh] OR infant* OR newborn* OR neonat* OR small for gestational age OR "low birth weight" OR premature AND (("maternity waiting home" OR "maternity waiting homes” OR "maternity waiting house” OR "maternity waiting houses” OR "maternal home" OR "maternal homes" OR “maternal house” OR "maternal houses”)) OR (((maternity OR maternal OR birth OR childbirth) PRE/3 (waiting OR shelter OR shelters OR hut OR huts)))

**Appendix II: Appraisal instrument**

**JBI critical appraisal checklist for cohort/case control studies**


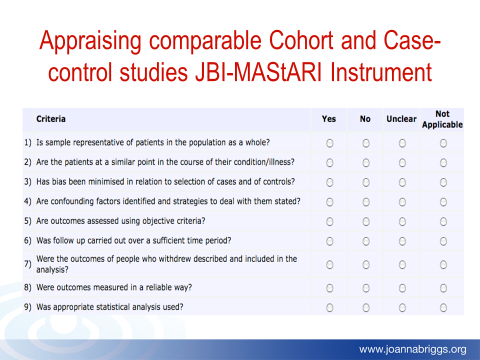


**Appendix III**

**JBI Data extraction format**


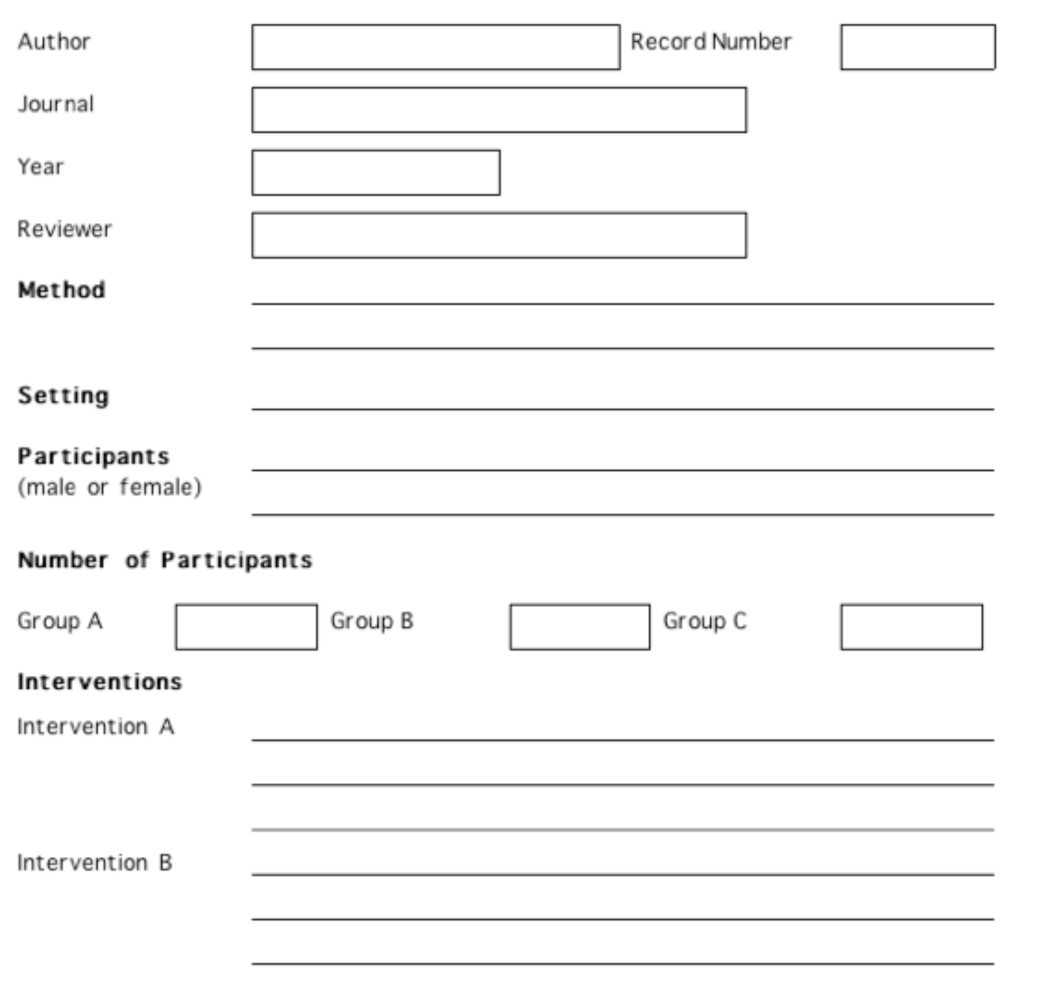

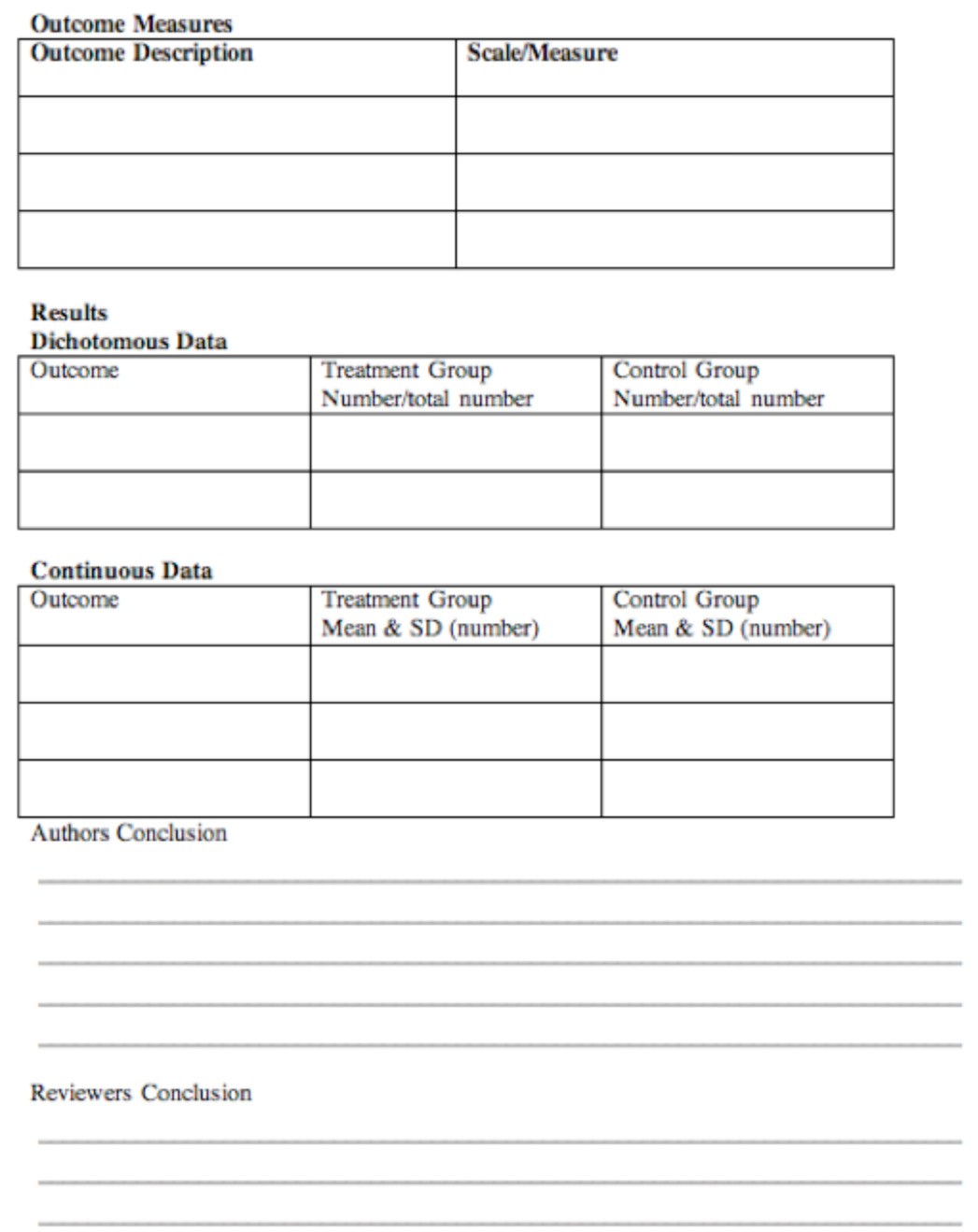

Supplement: Supplementary file 1 — This file contain Index and mesh terms, the search strategy used in different databases, critical appraisal formats, and data extraction format (DOCX 645 kb) [file 12913_2018_3559_MOESM1_ESM.docx]
